# Metabolic reprogramming through PIM3 inhibition reverses hypoxia-induced CAR-T cell dysfunction in solid tumors

**DOI:** 10.1186/s12967-025-07278-5

**Published:** 2025-11-06

**Authors:** Muya Zhou, Luxia Xu, Jinhua Hu, Wenwen Chen, Jialu Hong, Mufeng Wang, Zhigang Guo

**Affiliations:** 1 Jiangsu Key Laboratory for Molecular and Medical Biotechnology, College of Life Sciences, Nanjing Normal University, 1 WenYuan Road, Nanjing, 210023 China; 2 Nanjing CalmHome Institute of Cell and Gene Engineering, Nanjing, 210023 China

**Keywords:** Chimeric antigen receptor (CAR)-T cell therapy, Tumor microenvironment, Hypoxia, PIM3, Solid tumor

## Abstract

**Background:**

Chimeric antigen receptor (CAR)-T cell therapy has shown remarkable success in hematologic malignancies but faces significant challenges in solid tumors due to the immunosuppressive tumor microenvironment (TME). Among these, hypoxia plays a vital role, yet the molecular mediators that link hypoxia to CAR-T dysfunction remain incompletely understood.

**Methods:**

Anti-mesothelin (MSLN) CAR-T cells were cultured under normoxic (21% O_2_) and hypoxic (1% O_2_) conditions for six days. We assessed cell expansion, phenotypes, cytotoxicity, and metabolic features. RNA sequencing was conducted to identify key gene expression changes induced by hypoxia. Findings were further validated using anti-CD70 CAR-T cells.

**Results:**

Hypoxia reduced CAR-T proliferation, increased apoptosis, lowered memory phenotypes, raised exhaustion, and weakened cytotoxicity in short-term and long-term assays. Transcriptomic and metabolic analyses showed metabolic reprogramming with increased glycolysis and reduced oxidative phosphorylation. Among the dysregulated genes, the serine/threonine-protein kinase PIM3 emerged as a previously underexplored mediator of hypoxia-driven dysfunction. Genetic or pharmacologic inhibition of PIM3 counteracted hypoxia-induced impairment, enhancing memory phenotypes of CAR-T cells, and improving their anti-tumor activity both in vitro and in vivo.

**Conclusions:**

This work identifies PIM3 as a previously underexplored target that links hypoxia to CAR-T cell dysfunction and demonstrates that PIM3 inhibition can reverse these effects. These findings provide a mechanistic rationale for incorporating PIM3 inhibition into CAR-T cell manufacturing or engineering to improve their therapeutic potential in hypoxic solid tumors.

**Supplementary Information:**

The online version contains supplementary material available at 10.1186/s12967-025-07278-5.

## Background

Chimeric antigen receptor (CAR)-T cell therapy has played a pivotal role in combating blood cancers, since its initial approval by the United States Food and Drug Administration (FDA) in 2017 [1]. As of now, six products targeting either CD19 or B-cell maturation antigen (BCMA) have received approval. The success of CAR T cells in the treatment of hematological malignancies has provided the impetus for investigating this approach in solid tumors [2, 3]. However, the application of CAR-T cell therapy in solid tumors has been significantly less effective. One critical factor contributing to this discrepancy is the immunosuppressive tumor microenvironment (TME), particularly the hypoxic conditions that prevail within solid tumors [4].

Due to the rapid and uncontrolled proliferation of tumor cells and the resulting increased diffusion distance from capillaries, chronic hypoxia develops within tumors, making it a typical characteristic of nearly all solid tumor microenvironments [5–8]. In the solid tumor microenvironment, the host immune response to tumor cells is shaped by hypoxic conditions. Through upregulating CD47 expression on tumor cells, hypoxia prevents macrophage-mediated phagocytosis [9, 10] and activates autophagy in tumor cells, reducing their sensitivity to cytotoxic T lymphocyte (CTL)- and natural killer (NK) cell-mediated cytotoxicity [11, 12]. Hypoxia also promotes the expression of inhibitory factors on tumor cells to mitigate the functions of various immune cells [13–19], and induces the production of immunosuppressive metabolic products [20]. In addition, hypoxia can influence immune cells directly. As the central component of the immune system to kill tumor cells, the cytotoxic function of T cells is blocked under hypoxic conditions. Research indicates that hypoxia limits the efficacy of T cells in solid tumors by driving metabolic alterations, functional impairment, and growth arrest in T cell subsets [21–26]. Hypoxia-inducible factors (HIFs) are a family of transcription factors that specifically respond to hypoxia. HIFs have shown the potential to shift metabolism from oxidative phosphorylation to glycolysis, leading to energy inefficiency and accumulation of inhibitory by-products, which reduces T cell viability, proliferation and activity [27–29].

Given that CAR-T cells are derived from T cells, their function and efficacy are supposed to be affected by the hypoxic conditions within the TME in a similar way. Recent studies observed that hypoxia could impede the proliferation and killing capacity of CAR-T cells [30–32]. Addressing the challenges posed by hypoxia in the TME is necessary for improving CAR-T cell therapy in solid tumors. Modifications of the CAR structure have been investigated by several groups. By incorporating an oxygen-dependent degradation domain (ODD) of HIF1a into the CAR structure and modifying its promoter to include hypoxia-response elements (HREs), Kosti et al. developed a dual hypoxia-sensing system which enabled selective expression of CAR under hypoxia [30]. Furthermore, Zhou et al. developed a CD19 CAR-T cell expressing hypoxia-regulated IL-12 (CAR19/hIL12ODD). In this construct, IL-12 was fused with the ODD of HIF1a, so that it was only secreted within the hypoxic TME [31]. Zhu et al. employed a hypoxia-responsive 5H1P promoter, enabling CAR-T cells to be specifically activated in hypoxic environments [32]. So far, the impacts of hypoxia on the effectiveness of CAR-T cells in treating solid tumors have not been systematically studied, and the underlying mechanisms remain unclear. Moreover, there is a need for more strategies that can mitigate hypoxia-induced damage and enhance CAR-T cell therapy for solid tumors, with a focus on safety and broader availability in clinical application.

In this study, to investigate the impacts of hypoxia on CAR-T cells, we used a tri-gas incubator and compared CAR-T cells cultured under 21% O_2_ and 1% O_2_. We found that hypoxia induced apoptosis, terminal differentiation, exhaustion and excessive activation of CAR-T cells, and impaired their proliferation and cytokine secretion. The cytotoxicity of hypoxic CAR-T cells in both short-term and long-term models were reduced as well. To further explore the underlying mechanisms, we did transcriptome sequencing of normoxic and hypoxic CAR-T cells. The results showed that hypoxia induced differential expression of genes related to CAR-T cell function and metabolic reprogramming. We identified the serine/threonine-protein kinase PIM3 as a promising target for addressing hypoxia-induced damage through metabolism regulation. Inhibition of PIM3 effectively rescued CAR-T cells from the profound immunosuppressive effects of hypoxia, resulting in enhanced tumor-killing activity and prevention of recurrence in a solid tumor mouse model. This line of investigation holds promise for extending the remarkable success of CAR-T cell therapy from hematologic malignancies to solid tumors.

## Methods

### Cell lines and culture

Human ovarian cancer cell lines OVCAR3 and SKOV3, cervical carcinoma cell line HeLa, glioblastoma cell line U87, clear cell renal cell carcinoma cell line 786-0, breast cancer cell line HCC1806, and embryonic kidney 293 T cells (HEK293T) were purchased from the American Type Culture Collection (ATCC). The MSLN-overexpressing and firefly luciferase (ffluc)-expressing SKOV3-MSLN-GFP-luc cells, as well as other tumor cell lines that express ffluc, were obtained through lentiviral transduction. HEK293T cells were cultured in DMEM medium (Gibco) supplemented with 10% FBS. OVCAR3-luc, HCC1806-luc and HeLa-luc cells were cultured in RPMI1640 medium (Gibco) supplemented with 10% fetal bovine serum (FBS; Gibco). SKOV3-MSLN-luc cells were cultured in McCoy’s 5 A medium (Gibco) supplemented with 10% FBS. Cells were culture in a 37 ℃ incubator with 5% CO_2_ supplementation. Hypoxic incubation was performed using a tri-gas incubator (BOLV INSTRUMENT, POU-90 A) set to 1% O_2_ and 5% CO_2_ at 37 °C to mimic tumor hypoxia, since oxygen levels in tumor tissues are usually 1% O_2_ or less [33].

### The generation of CAR-T cells

The generation of CAR-T cells was carried out according to the methodology described in our previous studies [34, 35]. CAR constructs targeting MSLN or CD70 were developed using fully human scFvs screened by our lab before, a CD8α signaling peptide, hinge and transmembrane regions, and an intracellular CD28/CD3ζ signaling domain, all cloned into the pCDH lentiviral vector. Human T cells were isolated from peripheral blood mononuclear cells (PBMC; HYCELLS, hPB050C) using the EasySep™ Human T-Cell Isolation Kit (Stemcell, 17951). Activation was achieved with Dynabeads™ Human T-Expander CD3/CD28 beads (Thermo Fisher Scientific, 11141D) at a 1:1 ratio. The activated T cells were then transduced with the CAR-expressing lentivirus produced by HEK293T cells to generate CAR-T cells targeting MSLN. These CAR-T cells were cultured in X-VIVO medium (LONZA) supplemented with IL-2 (100 IU/mL). Fresh medium was added every 2–3 days to maintain a cell density of 1–2 × 10^6^/mL.

### Flow cytometry

The antibodies used in this study, including PE-anti-CD4, APC-anti-CD8, PerCP-7-AAD, PE-Annexin V, APC-anti-PD-1, FITC-anti-LAG-3, PE-anti-CD62L, APC-anti-CD45RO, APC-anti-CD69, PE-anti-CD25, APC-anti-CD107a, and PE-anti-IFN-γ, were all purchased from Biolegend. FITC-Labeled human MSLN protein was from KACTUS for human anti-MSLN CAR detection. For cell-surface staining, cells were washed with PBS after incubation for 15 min at room temperature and then all samples were examined using a CytoFLEX S (Beckman Coulter), and data were analyzed using CytExpert software.

To evaluate CD107a expression on the cell surface as anindirect marker of degranulation, CAR-T cells were first stimulated with phorbol 12-myristate 13-acetate (PMA) and ionomycin for 4 h, and monensin was added after one hour of stimulation. APC anti-human CD107a antibody was added along with the addition of PMA and ionomycin. After incubation, cells were stained with an APC-conjugated anti-CD3 antibody, followed by flow cytometry analysis.

For intracellular cytokine staining, after treatment of PMA, ionomycin, and monensin afterwards, cells were permeabilized and fixed using the Cytofix/Cytoperm kit (BD Biosciences) according to the manufacturer’s instructions. Then CAR-T were incubated with fluorescent antibodies and examined via flow cytometry.

### Cytotoxicity assays

In the short-term cytotoxicity assay, CAR-T cells conditioned under normoxia or hypoxia were co-cultured with tumor cells at different effector-target (E: T) ratios for 24 h and cytotoxicity was detected. For multiple rounds of killing analysis, conditioned CAR-T cells were challenged with tumor cells at indicated E: T ratios for 24 h. After that, CAR-T cells were transferred and co-cultured with fresh tumor cells for another 24 h. Together, three rounds of co-culture were conducted and cytotoxicity of CAR-T cells at each round was examined.

The cytotoxicity of CAR-T cells was assessed using the One-Lite Luciferase Assay System (Vazyme, DD1203-02) according to the manufacturer’s instructions. The cell killing rate was calculated using the following formula: Cytotoxicity = (control value - experimental value) / control value × 100%.

### Spheroid killing assay

The green fluorescent protein (GFP)-expressing SKOV3-GFP cells were seeded in the ultra-low attachment round-bottom 96-well plate (Corning, 7007) with the number of 10,000 cells per well. Tumor spheroids were allowed to form and grow for 48 h. Then 10,000 WT CAR-T or PIM3 KO CAR-T cells were added each well and fluorescent images were captured via a fluorescent microscopy 24 h later. Tumor spheroids without the addition of CAR-T cells served as the control group. Fluorescence intensity of the tumor spheroids was measured using ImageJ and the killing efficiency of CAR-T cells was calculated by (control intensity - experimental intensity) / control intensity × 100%.

### Cytokine release detection

Secreted cytokine levels in CAR-T cell were examined by cytometric bead array (CBA) assay kit (BD Pharmingen, 558264) following the manufacturer’s instructions. Generally, multiple capture beads were mixed together, including TNF-α (BD Pharmingen, 558273), IL-10 (BD Pharmingen, 558274), IFN-γ (BD Pharmingen, 560111), granzyme B (BD Pharmingen, 560304). Cell culture or blood supernatants were incubated with mixed capture beads for 1 h, and then mixed with detection reagent for 2 h. The beads were washed carefully and resuspended in PBS. Samples were analyzed by flow cytometry and the obtained data were analyzed with FCAP Array software.

### Real-time quantitative polymerase chain reaction (qPCR)

Cells were collected and total RNA was isolated using the FastPure Cell/Tissue Total RNA Isolation Kit V2 (Vazyme, RC112). Reverse transcription was performed with the HiScript III 1st Strand cDNA Synthesis Kit (+ gDNA wiper) (Vazyme, R312) according to the manufacturer’s instructions. qPCR were conducted using the 2×Taq Pro Universal SYBR qPCR Master Mix (Vazyme, Q712). The qPCR primers are listed in Supplementary Table 1.

### Seahorse metabolic assays

Mitochondrial respiration and lactate secretion of T cells were assessed by measuring the oxygen consumption rate (OCR) and glycolytic proton efflux rate (glycoPER), respectively, using a Seahorse XFe96 extracellular flux analyzer (Agilent). XFe96 cell culture microplates (Agilent) were pre-coated with poly-D-lysine, and 2 × 10^5^ T cells in Seahorse XF RPMI medium (Agilent) supplemented with 2 mM L-glutamine (Gibco), 1 mM sodium pyruvate (Sigma), and 10 mM D-glucose (Sigma) were seeded in each well. Following one hour of incubation in a CO_2_-free incubator at 37 °C, glycolytic and mitochondrial stress tests were conducted as per the manufacturer’s protocol.

### RNA-sequencing

CAR-T cells were cultured under normoxic or hypoxic conditions for 6 days. Live cells were sorted using a BD FACSAria™ III (BD Biosciences). Subsequently, cells were collected, resuspended in TRIzol and sent for RNA-sequencing (RNA-seq), which was conducted by Gene Denovo Co., Ltd.

### PIM3 knockout (KO)

PIM3 KO was conducted using the CRISPR/Cas9 system as follows: An RNP complex was prepared through mixing 60 pmol of Cas9 protein and 100 pmol of sgRNA (sequences listed in Table S2; Beijing Zeping), followed by incubation at room temperature for 10 min. Two million CAR-T cells were resuspended in electroporation buffer and mixed with the RNP complex before being electroporated using a Lonza nucleofector. Following electroporation, the cells were immediately diluted in pre-warmed cell culture medium and incubated at 37 °C for 15 min. The cell suspension was then transferred to a 12-well plate for culture. After 48 h, the cells were moved to a 6-well plate and maintained at a concentration of 1 × 10^6^ cells/ml, with medium replenished every 48 h.

### Animal experiments

Animal experiments were conducted at the animal laboratory of Nanjing Normal University in accordance with protocols approved by the Animal Welfare Committee of the university (Approval number: 2020-0047). To establish the subcutaneous tumor model, 7-week-old C-NKG mice were obtained from Cyagen Biosciences. Two million 1806 cells were injected subcutaneously in each mouse. For CAR-T treatment, 100 µl of 1 × 10^6^ CAR-T cells each mouse were injected via the tail vein 12 days post tumor inoculation. Tumor volumes and body weights were measured and recorded every other day, with tumor volume calculated as (length × width^2^)/2. Mice were sacrificed when the tumor volume reached around 1000 mm^3^. Mice blood and tissue samples were collected 10 days post CAR-T treatment, followed by flow cytometry, CBA and immunochemical analysis.

### Analysis of tissue samples

Immunohistochemical analysis of tissue samples was conducted by Servicebio. In breif, for hematoxylin-eosin (HE) staining, mice tissues were fixed in paraformaldehyde, processed into paraffin sections, stained with hematoxylin and eosin, and mounted with mounting medium. For immunohistochemical (IHC) staining, following fixation in paraformaldehyde, tumor tissues were embedded in paraffin and sectioned. The sections were then dewaxed, rehydrated, and rinsed with ddH_2_O. Afterwards, antigen retrieval was conducted, followed by cooling and washing with PBS. The sections were then blocked with 3% BSA and incubated overnight at 4 °C with the anti-CD45 human primary antibody. After washing with PBST, the sections were incubated with the secondary antibody, and then counterstained with hematoxylin, and mounted with a coverslip using mounting medium. Images were captured through a slide scanner and analyzed using ImageJ.

### Statistical analysis

All statistical calculations and graphs were generated by GraphPad Prism 10.1.2. For statistical analysis, two-tailed unpaired t-test, one-way analysis of variance (ANOVA) and two-way ANOVA was used. A P-value < 0.05 was considered statistically significant. Results are presented as mean ± SD unless otherwise indicated.

## Results

### Hypoxia impaired CAR-T cell survival, proliferation, memory phenotypes, and cytotoxic function

**Fig. 1:**
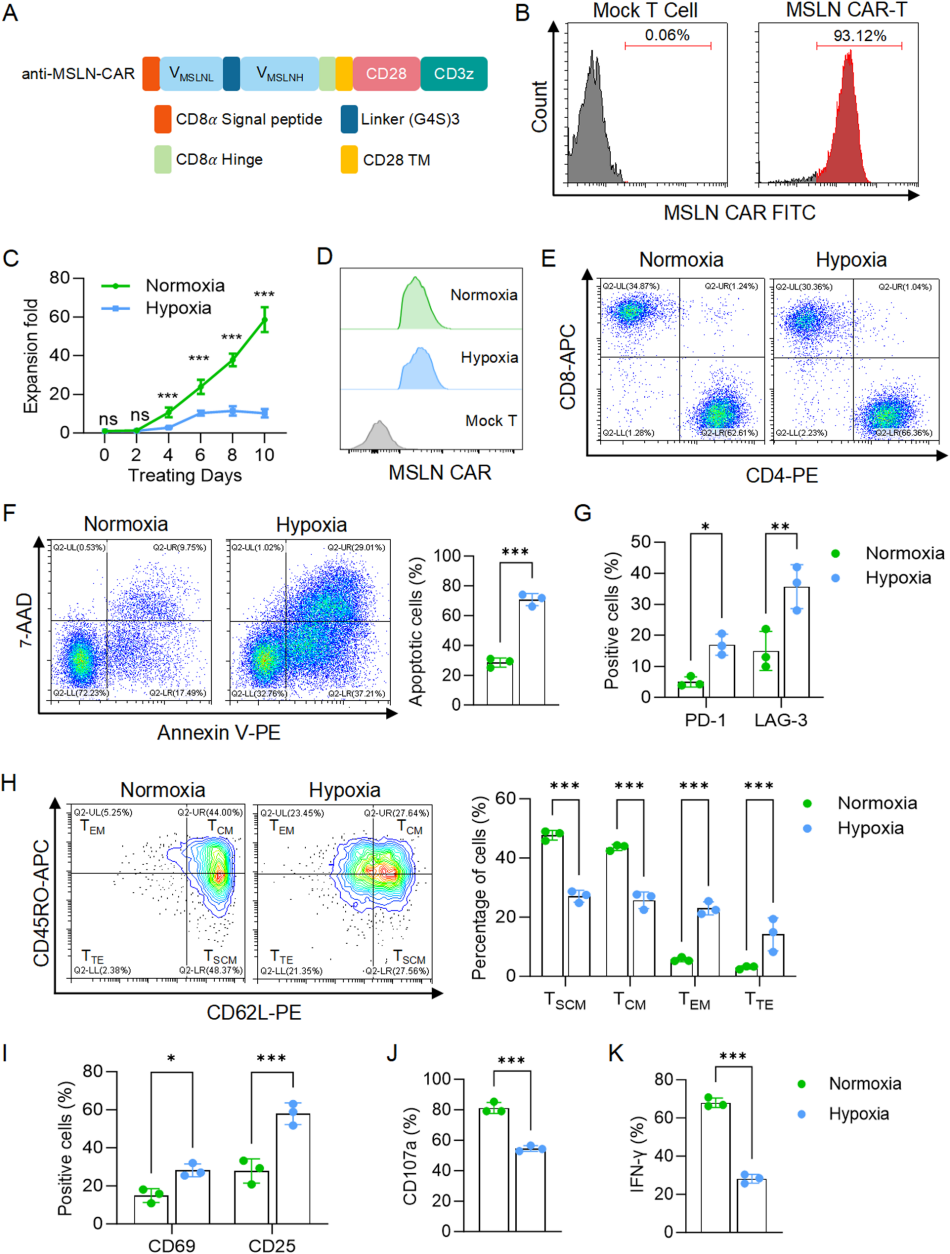
Hypoxia impaired the survival, proliferation and memory phenotypes of anti-MSLN CAR-T cells. **A**, Schematic representation of the anti-MSLN CAR-T structure. **B**, Histograms showing the CAR expression rate in non-transduced human T cells and anti-MSLN CAR-T cells. **C**, Six days after T-cell isolation and activation, equal numbers of CAR-T cells from three independent donors were subjected to normoxic or hypoxic culture. The expansion fold was calculated relative to the cell number at the start of treatment. **D**, Histograms of CAR expression in normoxic and hypoxic CAR-T cells. **E**, Representative flow cytometry plots showing the expression of CD4 and CD8 in CAR-T cells after 6 days of normoxic or hypoxic treatment. **F**, Representative flow cytometry plots and quantification results indicating the apoptotic rate of CAR-T cells after 6 days of normoxic or hypoxic treatment. Apoptotic cells are defined as Annexin V-postive. **G**, Expression of PD-1 and LAG-3 in CAR-T cells under normoxic or hypoxic conditions. **H**, Representative flow cytometry plots and quantification results indicating the changes in differentiation status of CAR-T cells after 6 days of exposure to normoxia or hypoxia. T_SCM_, stem cell memory T cells, CD62L + CD45RO-. T_CM_, central memory T cells, CD62L + CD45RO+. T_EM_, effector memory T cells, CD62L-CD45RO+. T_TE_, terminal effector T cells, CD62L-CD45RO-. **I**, Expression of CD69 and CD25 in CAR-T cells under normoxic or hypoxic conditions. **J**, Surface expression of CD107a in CAR-T cells conditioned under normoxia or hypoxia, upon stimulation with PMA and ionomycin. **K**, Intracellular flow cytometric analysis showing IFN-γ expression in conditioned CAR-T cells. All data are from at least three donors and are presented as mean ± SD. ns, not significant; **p < 0.01; ***p < 0.001, determined by two-way analysis of variance (ANOVA) (**C**, **G**, **H** and **I**) and two-tailed unpaired t-test (**F**, **J** and **K**)

To investigate the effects of hypoxia on CAR-T cells, we generated anti-mesothelin (MSLN) CAR-T cells using a validated CAR sequence from our laboratory (Fig. 1A-B) and cultured them under normoxic (21% O_2_) or hypoxic (1% O_2_) conditions. Under hypoxia, CAR-T cell proliferation was significantly inhibited, with no expansion observed after six days (Fig. 1C). Based on this, we selected a six-day hypoxia exposure period for subsequent experiments, ensuring that hypoxia-induced changes were significant while maintaining cell viability. Despite hypoxia treatment, MSLN CAR expression and the CD4/CD8 ratio remained unchanged (Fig. 1D-E). Analysis of hypoxic CAR-T cells revealed multiple functional impairments. A higher proportion of apoptotic cells (Annexin V+) was observed under hypoxia compared to normoxia (Fig. 1F). In the absence of antigen stimulation, hypoxic CAR-T cells exhibited increased expression of the exhaustion markers PD-1 and LAG-3 (Figs. 1G). Hypoxia also reduced the frequencies of stem cell memory (T_SCM_) and central memory T cells (T_CM_) subsets while increasing effector memory (T_EM_) and terminal effector (T_TE_) populations (Fig. 1H). In addition, hypoxic CAR-T cells exhibited heightened activation, as indicated by elevated CD69 and CD25 expression (Fig. 1I). Upon PMA and ionomycin stimulation, hypoxia-conditioned CAR-T cells showed significantly lower CD107a and IFN-γ levels compared to normoxic cells, indicating impaired degranulation and cytokine secretion (Fig. 1J-K). Overall, these findings suggest that hypoxia compromises CAR-T cell viability, proliferation, memory, and functional phenotypes, ultimately limiting their therapeutic potential in solid tumors.

### Hypoxia reduced both short-term and long-term anti-tumor efficacy of CAR-T cells

**Fig. 2:**
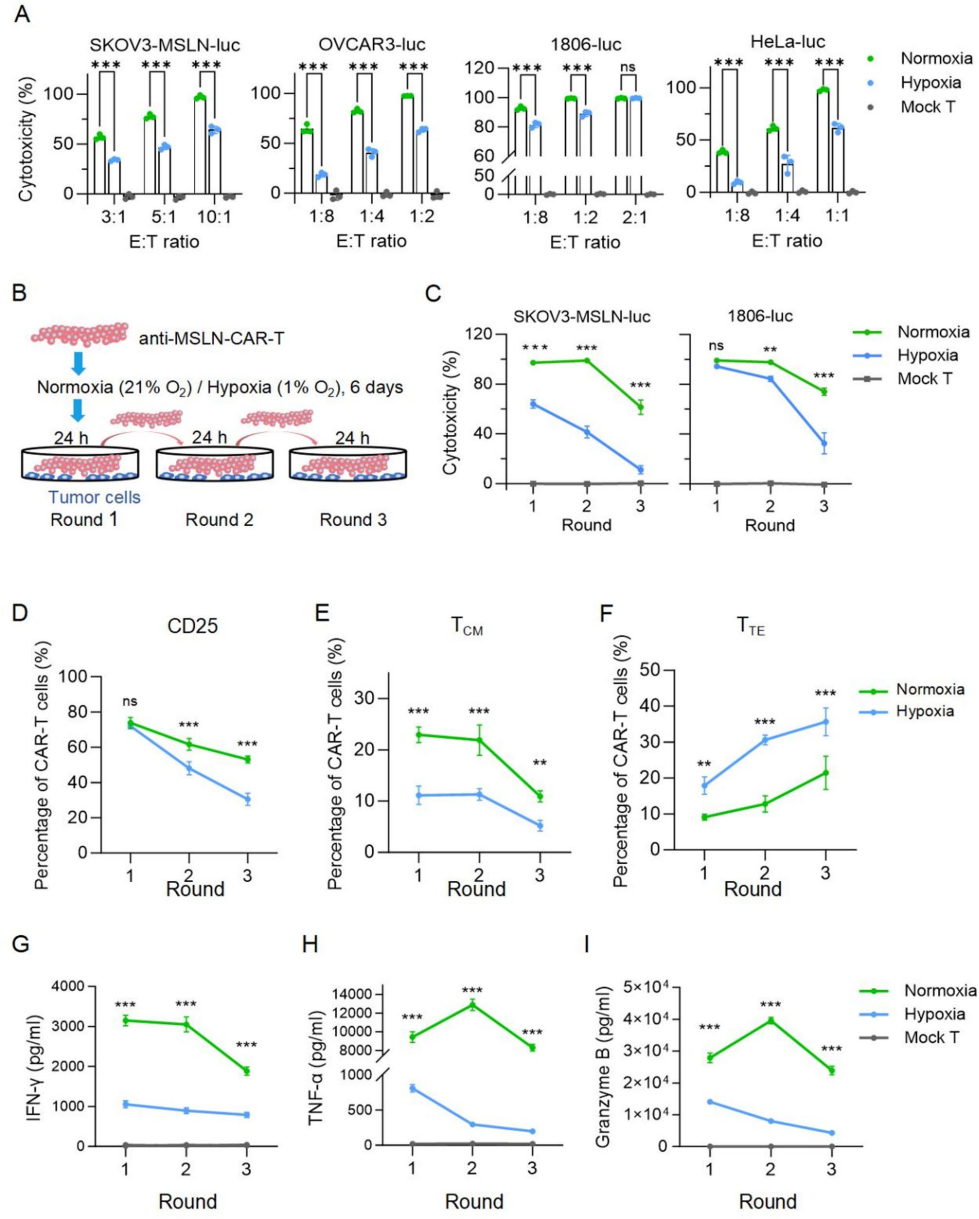
Hypoxia impaired the short-term and long-term killing efficacy of anti-MSLN CAR-T cells. **A**, CAR-T cells cultured under normoxic or hypoxic conditions were co-cultured with four target cell lines (SKOV3-MSLN-luc, OVCAR3-luc, 1806-luc, HeLa-luc) at the indicated effector-to-target (E: T) rations for 24 h under normoxia, and the killing efficacy was detected by luciferase reporter assay. Mock T, untransduced T cells. **B**-**C**, Multiple rounds of killing assays of conditioned CAR-T cells against SKOV3-MSLN-luc cells at an E: T ratio of 10:1 and 1806-luc cells at an E: T ratio of 2:1 were performed under normoxia. **D**-**E**, Expression of activation (**D**) and memory (**E**-**F**) markers in CAR-T cells after each round of killing against SKOV3-MSLN-luc cells in (**C**). **G**-**I**, Supernatants were collected after each round of killing in (**C**), and the secretion of IFN-γ, TNF-α, and granzyme B from conditioned CAR-T cells was measured using Cytometric Bead Array (CBA). All data are from at least three donors and are presented as mean ± SD. ns, not significant; *p < 0.05; **p < 0.01; ***p < 0.001, determined by two-way ANOVA (**A**, **C**, **D**-**F**)

To assess the impact of hypoxia on CAR-T cell cytotoxicity, we co-cultured normoxia- or hypoxia-conditioned CAR-T cells with four tumor cell lines (SKOV3-MSLN-luc, OVCAR3-luc, 1806-luc, and HeLa-luc) at specified effector-to-target (E: T) ratios for 24 h under normoxic conditions. Hypoxia-preconditioned CAR-T cells exhibited significantly impaired cytotoxicity across all tumor models (Fig. 2A), consistent with the observed reduction in CD107a and IFN-γ levels (Fig. 1J-K). To further evaluate long-term cytotoxicity, we conducted a multiple-round tumor-killing assay (Fig. 2B). Upon repeated antigen stimulation with SKOV3-MSLN-luc and 1806-luc cells, hypoxia-treated CAR-T cells exhibited markedly diminished long-term cytotoxicity (Fig. 2C).

Phenotypic analysis following each round of tumor stimulation indicated that hypoxia-conditioned CAR-T cells had a decreased proportion of activated and central memory phenotype cells (T_CM_) (Fig. 2D-E), while terminal effector phenotype cells (T_TE_) were significantly increased (Fig. 2F). Additionally, cytokine analysis showed reduced secretion of IFN-γ, TNF-α, and granzyme B in hypoxic CAR-T cells (Fig. 2G-I). Together, these findings indicate that hypoxia severely impairs CAR-T cell cytotoxicity, especially long-term cytotoxicity, and functional phenotypes during the tumor-killing process.

### Hypoxia induced differential expression of function-related genes and metabolic reprogramming in CAR-T cells

**Fig. 3:**
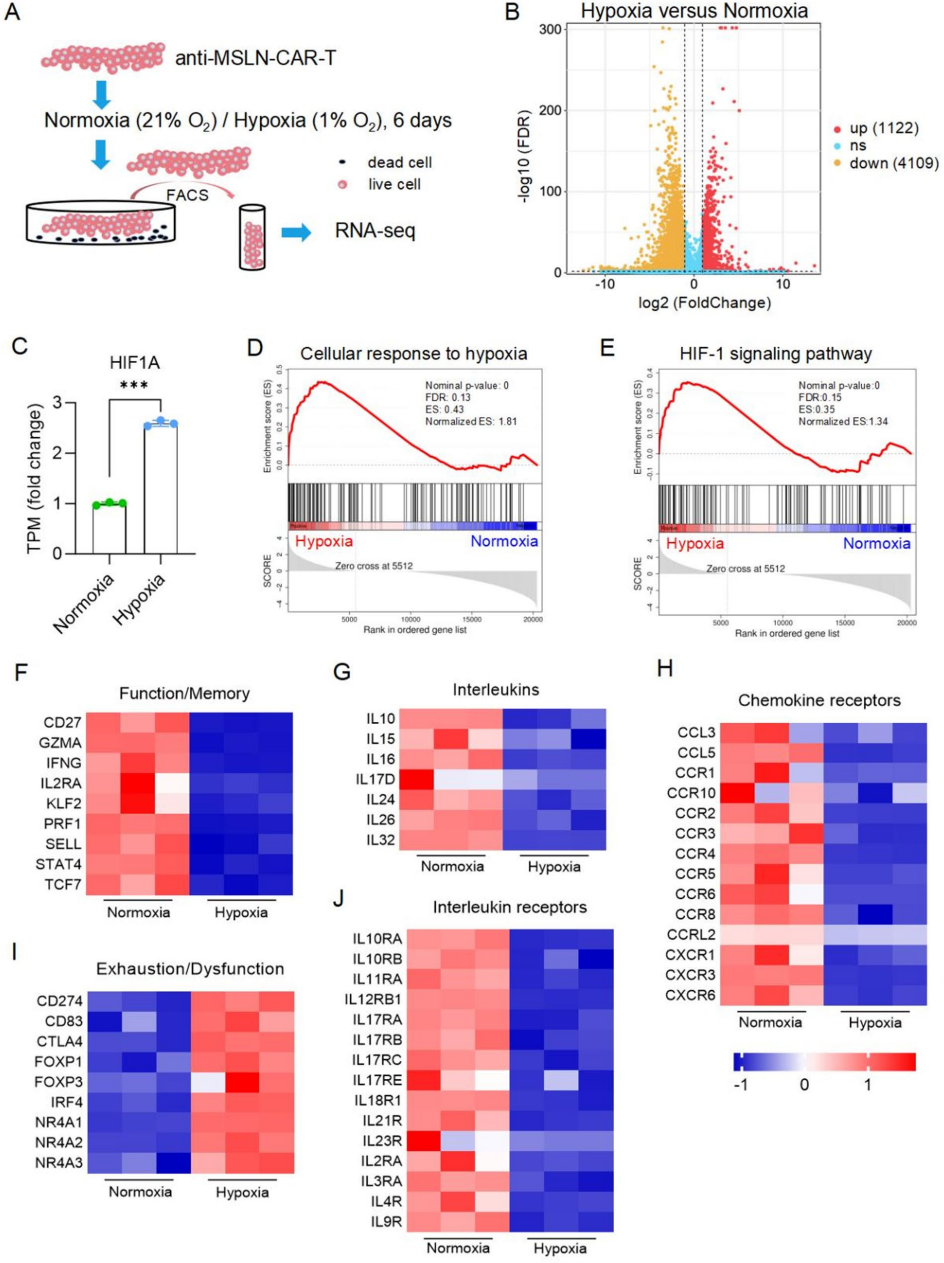
Hypoxia induced differential expression of function-related genes in CAR-T cells. **A**, CAR-T cells were cultured under normoxic or hypoxic conditions for 6 days. Live cells (negative for both 7-AAD and Annexin V) were sorted via FACS and subsequently subjected to RNA sequencing. **B**, Volcano plot depicting the differentially expressed genes (DEGs) under hypoxic versus normoxic conditions. **C**, Expression of hypoxia-inducible factor 1-alpha (HIF1a) was significantly increased in cells after hypoxic treatment. **D**-**E**, Gene set enrichment analysis (GSEA) revealed enrichment of genes involved in cellular responses to hypoxia and the HIF-1 signaling pathway in hypoxic CAR-T cells. **F**-**J**, Heatmaps illustrating DEGs related to CAR-T cell immune responses. The color bar indicates normalized z-scores for each DEG. ***p < 0.001, determined by two-tailed unpaired t-test (**C**)

To explore the mechanisms underlying hypoxia-induced CAR-T cell dysfunction, we performed RNA sequencing on live CAR-T cells sorted via flow cytometry after six days of normoxic or hypoxic culture (Fig. 3A). Hypoxia-treated CAR-T cells exhibited extensive gene expression alterations (Fig. 3B), including significant upregulation of hypoxia-related genes such as HIF1A (Fig. 3C). Gene set enrichment analysis (GSEA) further confirmed that hypoxia strongly activated hypoxia-responsive pathway and the HIF-1 signaling pathway (Fig. 3D-E). Additionally, consistent with our previous findings, hypoxia downregulated genes associated with CAR-T cell functionality and memory phenotypes, as well as genes encoding interleukins, interleukin receptors and chemokine receptors, while upregulating genes linked to exhaustion and dysfunction (Fig. 3F-J). These findings provide a molecular basis for the impaired anti-tumor function of CAR-T cells under hypoxic conditions.

**Fig. 4:**
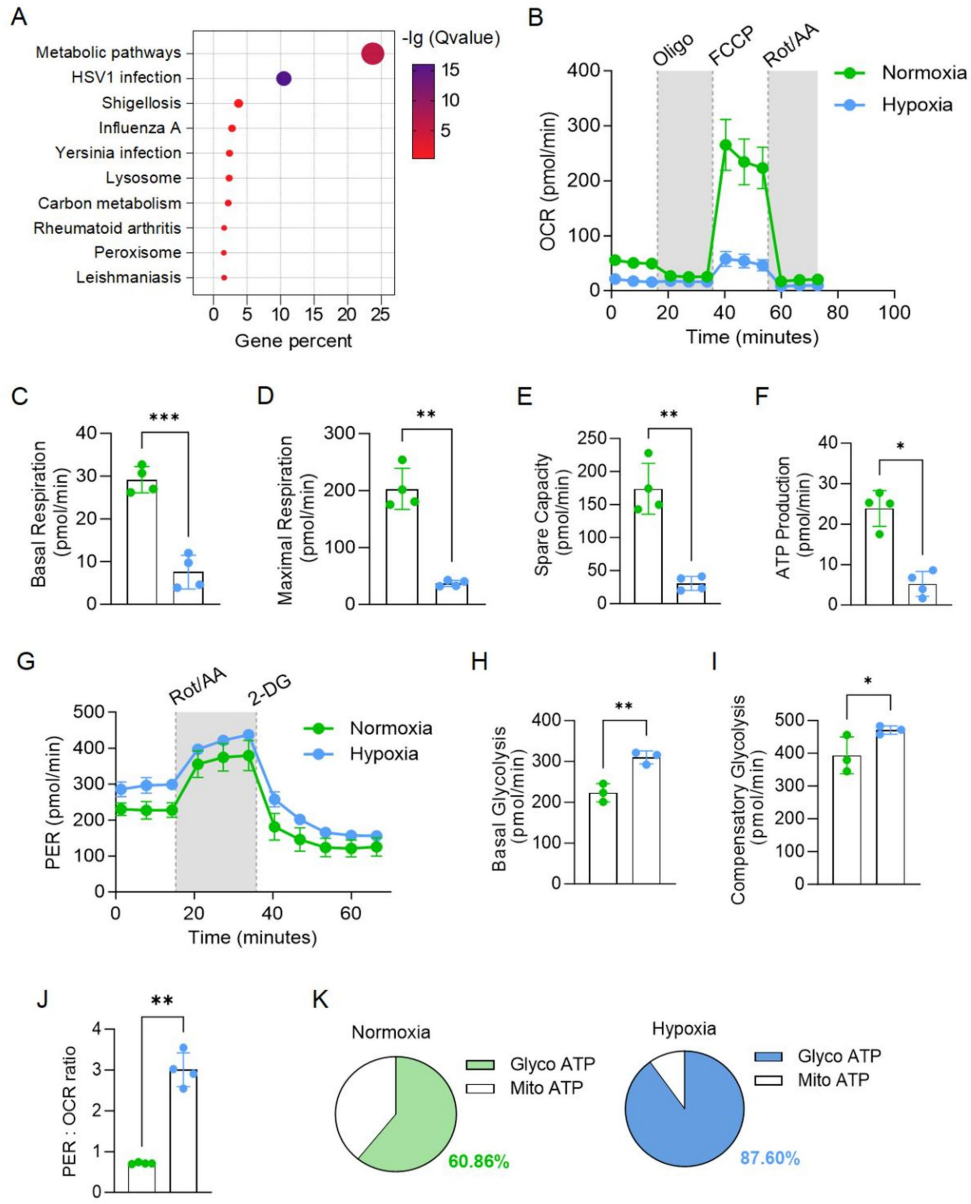
Hypoxia induced metabolic reprogramming of CAR-T cells by promoting glycolysis and inhibiting oxidative phosphorylation. **A**, KEGG orthology (KO) analysis of DEGs in hypoxic versus normoxic CAR-T cells. **B**, Oxygen consumption rate (OCR) of CAR-T cells under normoxic or hypoxic conditions, measured by Seahorse analysis. **C**-**F**, Basal (**C**), maximal (**D**) and spare (**E**) OCR, and ATP production (**F**) in normoxic and hypoxic CAR-T cells. **G**, Proton efflux rate (PER) of CAR-T cells under normoxic or hypoxic conditions, measured by Seahorse analysis. **H**-**I**, Basal (**H**) and compensatory (**I**) glycolysis in normoxic and hypoxic CAR-T cells. **J**, Ratio of PER to OCR in normoxic and hypoxic CAR-T cells. **K**, Relative contribution of glycolysis and mitochondrial respiration to cellular ATP production in normoxic and hypoxic CAR-T cells. All data are from at least three donors and are presented as mean ± SD. *p < 0.05; **p < 0.01, determined by two-tailed unpaired t-test (**C**-**F**, **H**-**J**)

Kyoto Encyclopedia of Genes and Genomes (KEGG) pathway analysis revealed that metabolic pathways were significantly altered in hypoxic CAR-T cells (Fig. 4A). Specifically, glycolysis-related genes were upregulated (Figure S1A), which has been linked to CAR-T cell exhaustion and terminal differentiation [36]. In contrast, genes involved in oxidative phosphorylation (OXPHOS), fatty acid oxidation, and mitochondrial carrier functions were downregulated (Fig. S1B-D).

To confirm these metabolic alterations, we measured the oxygen consumption rate (OCR) and proton efflux rate (PER) using Seahorse XF assays. OCR reflects OXPHOS efficiency and mitochondrial function, while PER quantifies glycolysis-driven proton release. Memory T cells are typically characterized by enhanced OXPHOS and reduced glycolysis [37, 38]. Under hypoxic conditions, CAR-T cells exhibited a significant reduction in mitochondrial respiration, as indicated by decreased basal respiration, maximal respiration, spare capacity, and ATP production (Fig. 4B-F). In contrast, both basal and compensatory glycolysis were upregulated in hypoxia-conditioned CAR-T cells (Fig. 4G-I). The increased PER to OCR ratio further confirmed a metabolic shift toward glycolysis in response to hypoxia (Fig. 4J). Additionally, decrease in MitoATP (OXPHOS-derived ATP) and an increase in GlycoATP (glycolysis-derived ATP) further highlighted this metabolic reprogramming (Fig. 4K). These findings suggest that hypoxia shifts CAR-T metabolism toward glycolysis, which contributes to their exhaustion and dysfunction.

### PIM3 inhibitor AZD1208 reversed hypoxia-induced damage and enhanced CAR-T cell efficacy

**Fig. 5:**
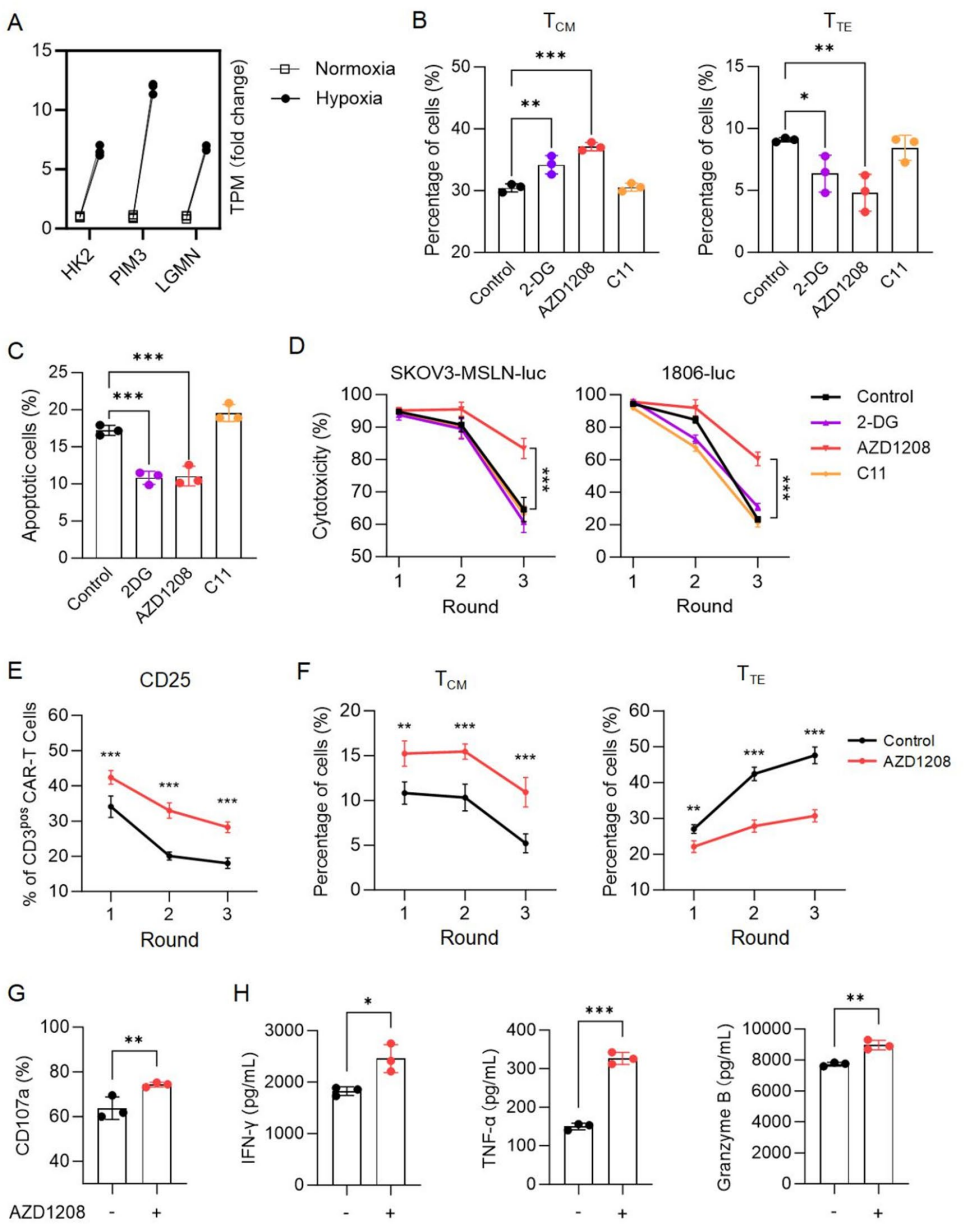
AZD1208 reversed hypoxia-induced damage and enhanced CAR-T cell efficacy. **A**, Differential expression of key genes including LGMN, PIM3 and HK2. **B**-**D**, CAR-T cells were cultured under hypoxic conditions for 6 days with single addition of 2-DG (2 mM), AZD1208 (2 µM), or C11 (1 µM). The memory phenotypes of CAR-T cells (**B**), as well as their apoptotic rates (**C**) were assessed by flow cytometry. Multiple rounds of killing assays (**D**) were performed under normoxia using conditioned CAR-T cells against 1806-luc cells at an E: T ratio of 2:1 and SKOV3-MSLN-luc cells at an E: T ratio of 10:1. **E**-**F**, Expression of activation (**E**) and memory (**F**) markers in CAR-T cells after each round of killing against SKOV3-MSLN-luc cells in (**D**). **G**, Surface expression of CD107a in CAR-T cells conditioned under hypoxia, upon stimulation with PMA and ionomycin. **H**, Supernatants were collected after round 3 of killing in (**D**), and the secretion of IFN-γ, TNF-α, and granzyme B from conditioned CAR-T cells was measured using CBA. All data are from at least three donors and are presented as mean ± SD. ns, not significant; *p < 0.05; **p < 0.01; ***p < 0.001, determined by one-way ANOVA (**B**-**C**), two-way ANOVA (**D**-**F**) and two-tailed unpaired t-test (**G**-**H**)

To identify potential therapeutic targets for reversing hypoxia-induced CAR-T dysfunction, we selected three significantly upregulated genes—LGMN, PIM3, and HK2—based on their significant upregulation in hypoxic CAR-T cells and literature-reported roles in glycolysis and immune suppression (Fig. 5A). Legumain (LGMN) is an asparaginyl-specific cysteine endopeptidase that has been identified as a hypoxia-responsive gene and the combined use of LGMN inhibitor and PD-1 inhibitor could enhance anti-tumor efficacy [39, 40]. While both provirus-integrating moloney site 3 (PIM3) and hexokinase 2 (HK2) have been reported to promote glycolysis, and their inhibitors could facilitate immunotherapies [41–44]. The upregulation of these genes was well validated through qPCR analysis (Figure S2A-C).

To confirm whether inhibiting these genes can reverse hypoxia-induced damage on CAR-T cell efficacy, we used inhibitors for each molecule: C11 for inhibiting LGMN, AZD1208 as a pan inhibitor of PIM kinases to inhibit PIM3, and 2-DG as an hexokinase inhibitor to inhibit HK2. CAR-T cells were exposed to hypoxic conditions with the respective inhibitors added and subsequent flow cytometric analysis and cytotoxicity measurement were conducted. Flow cytometry results revealed that after 6 days of hypoxia, the addition of 2-DG and AZD1208 significantly increased the central memory phenotype (T_CM_) and reduced the effector phenotype (T_TE_), with AZD1208 exhibiting the strongest effect (Fig. 5B). Moreover, 2-DG and AZD1208 reduced hypoxia-induced apoptosis in CAR-T cells compared to the untreated control group, while C11 exhibited no significant impacts (Fig. 5C). In terms of anti-tumor efficacy, the results of the long-term killing assays demonstrated that AZD1208 significantly improved the sustained killing ability of CAR-T cells against both 1806-luc and SKOV3-MSLN-luc tumor cells (Fig. 5D). Further analysis showed enhanced activation (Fig. 5E), increased T_CM_ phenotype and reduced T_TE_ phenotype (Fig. 5F) in AZD1208-treated CAR-T cells upon stimulation, as well as upregulated CD107a expression (Fig. 5G) and elevated secretion of IFN-γ, TNF-α, and granzyme B (Fig. 5H), indicating improved functionality after AZD1208 treatment. Based on these findings, we identified that the PIM3 inhibitor AZD1208 has greater potential to reverse hypoxia-induced damage in CAR-T cells.

To provide more evidence for our conclusions, we constructed anti-CD70 CAR-T cells following our previous studies to validate the ability of AZD1208 to reverse hypoxia-induced damage and enhance CAR-T cell anti-tumor function (Figure S3A-B) [35, 45]. The elevation of PIM3 under hypoxic conditions was confirmed through qPCR (Figure S3C). Consistent with the results observed in anti-MSLN CAR-T cells, hypoxia significantly impaired the memory phenotypes in anti-CD70 CAR-T cells, indicated by the reduced expression level of the memory marker CD62L (Figure S3D). The apoptotic rate of cells was also increased upon hypoxia treatment (Figure S3E). When co-culturing with AZD1208, anti-CD70 CAR-T cells showed a significantly enhanced memory phenotype and reduced apoptosis under hypoxic conditions (Figure S3D-E). Subsequently, both short-term and long-term cytotoxicity assays against U87-luc and 786-0-luc cells were performed and the results demonstrated that with the existence of AZD1208, the impaired short-term (Figure S3F) and long-term (Figure S3G-H) tumor-killing capabilities of anti-CD70 CAR-T cells after hypoxia treatment were restored. Together, these results demonstrate that inhibiting PIM3 with AZD1208 effectively mitigates hypoxia-induced CAR-T cell dysfunction.

### PIM3 KO CAR-T cells could resist hypoxia-induced damage and exhibited potent anti-tumor activity both in vitro and in vivo

Since AZD1208 is an inhibitor of pan PIM kinases, including PIM1, PIM2 and PIM3, we further detected the levels of PIM1 and PIM2 in hypoxic CAR-T cells and found that the expression of PIM1 and PIM2 showed no significant changes upon hypoxia treatment (Figure S4A). To confirm the specific therapeutic potential of PIM3 inhibition, we utilized the CRISPR/Cas9 system to knockout PIM3 in CAR-T cells (Figure S4B). Two sgRNAs were designed and added separately or together in the system to generate PIM3 knockout (KO) CAR-T cells. Compared with unmodified wild-type (WT) CAR-T cells, PIM3 KO CAR-T cells exhibited a higher proportion of memory phenotypes and a lower proportion of T_TE_ cells after six days of hypoxia treatment (Figure S4C-E), as well as significantly improved tumor-killing persistence in long-term killing assays (Figure S4F). Among the three KO cells, simultaneously adding both sgRNAs displayed best effects in promoting the persistent tumor-killing ability of hypoxic CAR-T cells. Therefore, the combination of sgRNA#1 and #2 was used to generate PIM3 KO CAR-T cells in the following experiments.

**Fig. 6:**
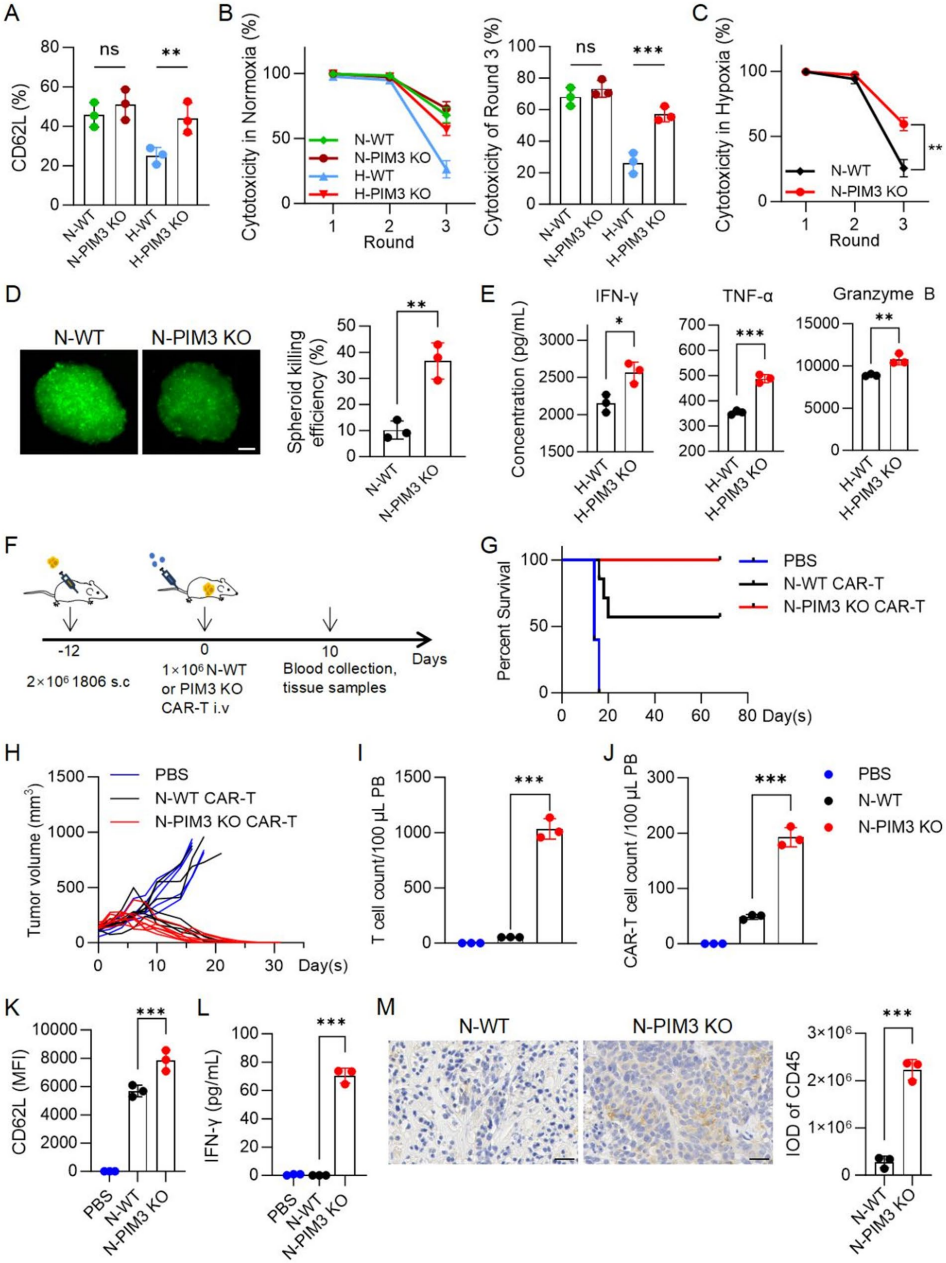
PIM3 KO CAR-T cells exhibited potent anti-tumor activity. **A**, CD62L expression of normoxia- or hypoxia-conditioend CAR-T cells. N-/H- refers to normoxic or hypoxic culture for 6 days. WT, wild-type (WT) CAR-T cells; PIM3 KO, PIM3 knockout (KO) CAR-T cells using both sgRNA#1 and #2. **B**, (left) Multi-round killing assays of WT and PIM3 KO CAR-T cells against 1806-luc cells conducted under normoxia. The right panel shows the cytotoxicy of Round 3. **C**, Multi-round killing assays of normoxia-cultured WT and PIM3 KO CAR-T cells against 1806-luc cells conducted under hypoxia. **D**, Representative fluorescent images of tumor spheroids 24 h post the addition of WT CAR-T or PIM3 KO CAR-T cells. Killing efficiency was calculated according to fluorescent intensity. Scale bar, 100 μm. **E**, Supernatants were collected after the third round of killing in (**B**), and the levels of IFN-γ, TNF-α, and granzyme B secreted by CAR-T cells were measured using a cytokine bead array (CBA). **F**, Schematic representation of the in vivo experimental model for PIM3 KO CAR-T cells. *N* = 5 mice for the PBS group, *N* = 7 mice for WT and PIM3 CAR-T groups. **G**, Survival analysis of mice. **H**, Tumor volume measurements of mice. I-K, Flow cytometric analysis of T cell (**I**) and CAR-T cell (**J**) subsets, and memory phenotype (**K**) in peripheral blood on day 10 post-treatment. **L**, The level of IFN-γ in blood supernatants on day 10 post-treatment was measured using CBA. **M**, IHC staining of CD45 was performed on tumor tissue sections. Scale bar, 25 μm. All data are from at least three individual experiments and are presented as mean ± SD. ns, not significant; *p < 0.05; **p < 0.01; ***p < 0.001, determined by one-way ANOVA (**A**, **B** and **I**-**L**), two-way ANOVA (**C**), two-tailed unpaired t-test (**D**, **E** and **L**)

Next, we compared the phenotypes and cytotoxicity of WT and PIM3 KO CAR-T cells under both normoxic and hypoxic treatment. The results showed that PIM3 KO slightly increased the expression of memory marker CD62L and long-term killing ability in normoxia-conditioned CAR-T cells, while the enhancement became much more significant in hypoxia-conditioned cells (Fig. 6A-B), indicating the crucial role of PIM3 inhibition in resisting hypoxia-induced damage. To further evaluate the cytotoxicity of PIM3 KO CAR-T cells in a hypoxic environment, we cultured CAR-T cells under normoxia and performed long-term cytotoxicity assays under hypoxia. In this hypoxic model, PIM3 KO CAR-T cells exhibited better long-term tumor-killing capacity as expected (Fig. 6C). Furthermore, we conducted the tumor spheroid killing assay, which used a three-dimensional model to mimic tumor hypoxia, and observed a much higher spheroid killing efficiency in the PIM3 KO group (Fig. 6D). In addition to improved phenotypes and tumor-killing efficiency, hypoxia-conditioned PIM3 KO CAR-T cells also secreted more IFN-γ, TNF-α, and granzyme B after being stimulated with tumor cells (Fig. 6E). Collectively, these results collectively suggest that knockout of PIM3 can mitigate hypoxia-induced damage on CAR-T cells and enhance their long-term cytotoxicity in vitro.

Next, we established a subcutaneous tumor model in C-NKG mice using 1806 cells to examine the anti-tumor efficacy of PIM3 KO CAR-T cells in vivo. Normoxia-cultured WT CAR-T or PIM3 KO CAR-T treatment was administered on day 12 post-tumor implantation, and blood, tissue, and tumor samples were collected on day 10 post-treatment for analysis. Tumor volumes, body weight changes, and survival status of the mice were continuously monitored and recorded (Fig. 6F). PIM3 KO CAR-T cells demonstrated superior tumor control, leading to complete tumor regression in all treated mice, whereas wild-type CAR-T cells achieved tumor clearance in only four out of seven mice (Fig. 6G-H). Flow cytometric analysis of blood samples revealed a higher proportion of CAR-T cells in the peripheral blood of mice treated with PIM3 KO CAR-T cells (Fig. 6I-J). These cells also exhibited an enhanced memory phenotype (Fig. 6K). Furthermore, CBA analysis indicated that PIM3 KO CAR-T cells secreted higher levels of IFN-γ in vivo (Fig. 6L). Immunohistochemical staining results exhibited increased PIM3 KO CAR-T cells in tumor tissues, which contributed to better tumor-eliminating rate in mice (Fig. 6M). In addition, hematoxylin-eosin (HE) staining of major tissues revealed no significant organ toxicity associated with PIM3 KO CAR-T cells (Figure S5A), and the overall body weights of the mice remained stable throughout the experiment, proving the safety of the modification (Figure S5B). Collectively, these findings confirm the strengthened solid tumor-killing ability of PIM3 KO CAR-T cells in vivo, which concurs with the observations in vitro, highlighting the potential of PIM3 inhibition as a promising strategy to overcome hypoxia-induced impairments.

### PIM3 KO CAR-T cells displayed increased OXPHOS and decreased glycolysis

**Fig. 7:**
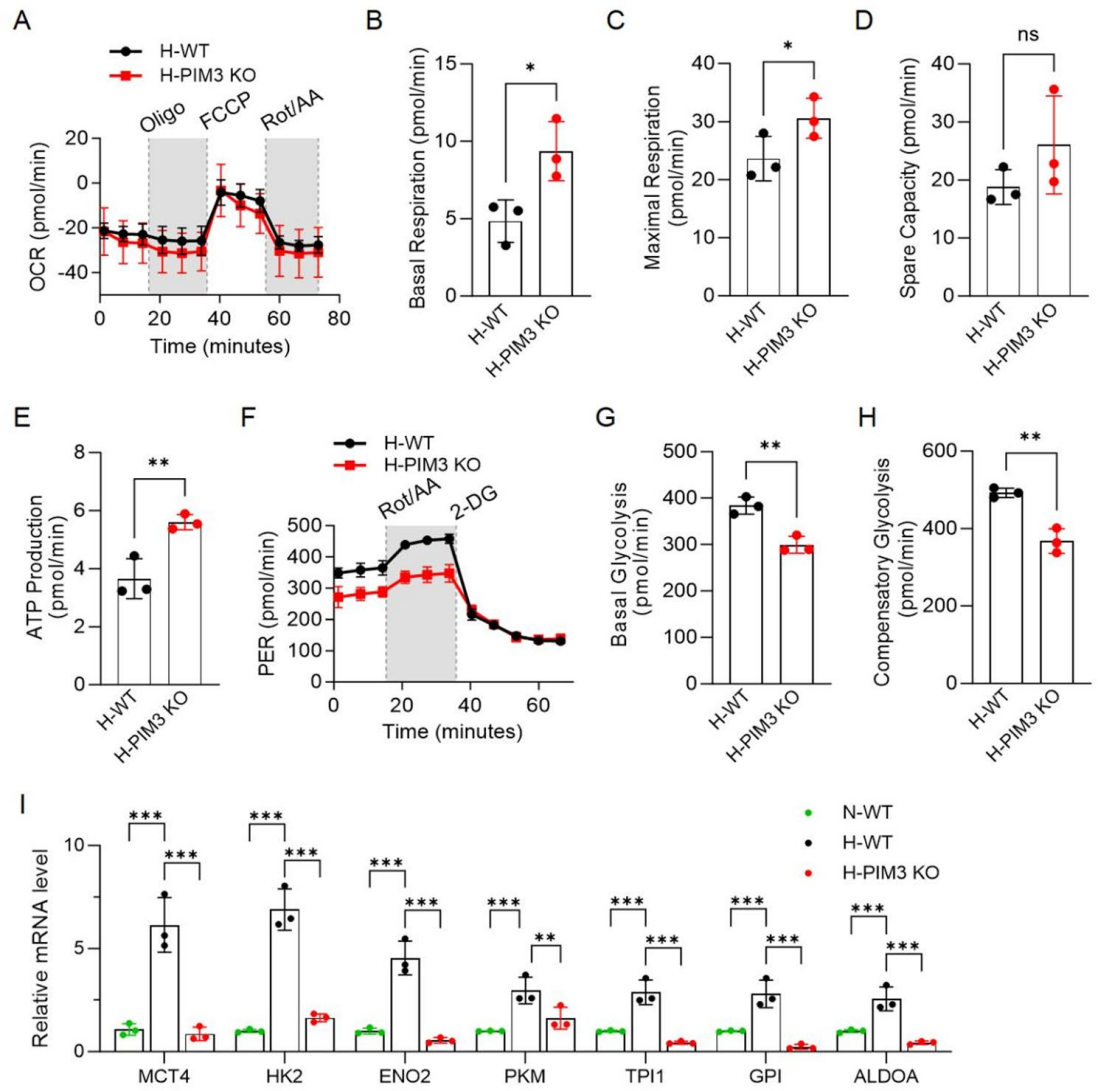
Metabolism of PIM3 KO CAR-T cells. **A**, OCR measurement of CAR-T cells using Seahorse analysis. **B**-**C**, Assessment of the basal OCR (**B**) and maximal OCR (**C**) of CAR-T cells. **D**, The spare respiratory capacity of CAR-T cells. E, The ATP production of CAR-T cells. **F**, PER measurement of CAR-T cells using Seahorse analysis. **G**-**H**, Evaluation of basal glycolysis (**G**) and compensatory glycolysis (**H**) of CAR-T cells. (**I**) Relative mRNA levels glycolysis-related genes in Normoxia-conditioned WT, hypoxia-conditioned WT and PIM3 KO CAR-T cells. All data are from at least three donors and are presented as mean ± SD. Ns, not significant; *p < 0.05; **p < 0.01; ***p < 0.001, determined by two-tailed unpaired t-test (**B**-**E**, **G**-**H**) and two-way ANOVA (**I**)

As previously demonstrated, hypoxic conditions induce metabolic reprogramming in CAR-T cells, which may well contributed to their functional impairments. Since PIM3 has been reported to regulate cell metabolism, we detected the OCR and PER levels in PIM3 KO CAR-T cells after being cultured under hypoxic conditions for six days to assessed whether PIM3 knockout influenced metabolic programming. The results showed that under hypoxic conditions, PIM3 KO CAR-T cells exhibited significantly enhanced OXPHOS activity, with elevated basal respiration, maximal respiration, spare capacity, and ATP production (Fig. 7A-E). In contrast, glycolysis levels were reduced, further supporting a shift toward OXPHOS metabolism (Fig. 7F-H). In addition, we measured the expression of glycolysis-related genes in hypoxia-conditioned PIM3 KO CAR-T cells. The results showed that the lactate transporter monocarboxylate transporter 4 (MCT4), as well as glycolytic genes including hexokinase 2 HK2, enolase 2 (ENO2), pyruvate kinase M1/2 (PKM), triosephosphate isomerase 1 (TPI1), glucose-6-phosphate isomerase (GPI), and aldolase, fructose-bisphosphate A (ALDOA), were elevated in hypoxic WT CAR-T cells but was significantly reduced upon PIM3 knockout (Fig. 7I). The downregulation of these glycolytic genes provides a mechanistic explanation for the impaired glycolytic flux and enhanced oxidative metabolism observed in PIM3 KO CAR-T cells. Collectively, these findings suggest that inhibition of PIM3 enhances CAR-T function through restoring mitochondrial metabolism and restricting the metabolic reprogramming from OXPHOS to glycolysis, thereby improving their memory phenotypes and therapeutic potential in hypoxic tumors.

## Discussion

The solid tumor microenvironment (TME) poses considerable challenges to CAR-T cell therapy, with hypoxia being one of the most critical barriers to effective tumor eradication [21, 46]. In our study, we utilized the 1% O_2_ condition to mimic the hypoxic environment, and observed that hypoxia severely impaired survival, proliferation, memory phenotypes, cytokine production, and anti-tumor cytotoxicity of both anti-MSLN and anti-CD70 CAR-T cells. Previous studies have also demonstrated the adverse impacts of hypoxia on T cells and CAR-T cells. Decreased proliferation, cytokine production and cytotoxicity were detected in both T and CAR-T cells [30, 32, 47, 48], while increased apoptosis and exhaustion were reported only in T cells [49]. Our findings systematically reveal the impacts of hypoxia on CAR-T cells and reinforce the need for strategies that can counteract hypoxia-induced suppression in CAR-T therapy for solid tumors.

Beyond functional impairment, we observed a metabolic shift in hypoxic CAR-T cells characterized by enhanced glycolysis and diminished oxidative phosphorylation, suggesting metabolic reprogramming in response to oxygen deprivation. This phenomenon has also been reported in T cells under hypoxic stress [22]. In line with the metabolic alterations that occurred in the hypoxic environment, metabolic regulators have displayed their potential to overcome hypoxia-induced damage and improve the therapeutic effects of CAR-T therapy in solid tumors. For instance, metformin has emerged as a promising molecule. Scharping et al. found that metformin could mitigate the hypoxic level within solid tumors and improve the efficacy of PD-1 blockade [50], while Finisguerra et al. showed that metformin facilitated adoptive cell therapy (ACT) through increasing the infiltration of T cells in hypoxic tumor tissues, rather than affecting tumor hypoxia [25]. Another study employed the combination of metformin and rapamycin during CAR-T cell culture, and this pretreatment benefited the anti-tumor efficacy of CAR-T cells in hypoxic environment [47], indicating the therapeutic promise of metabolic modulation.

In our study, we identified PIM3 kinase, which was significantly upregulated in hypoxic CAR-T cells, as a critical regulator of metabolic adaptation and CAR-T function under hypoxia. PIM kinases, including PIM1, PIM2, and PIM3, are serine/threonine kinases frequently overexpressed in tumors and contribute to cancer cell proliferation, survival, and metastasis [51–53]. Inhibitors targeting the PIM kinases have shown significant anti-tumor effects [54]. In recent years, the role of PIM kinases in immunomodulation are being investigated. For example, PIM kinases inhibit early human Th17 differentiation [55], and pan-PIM inhibition with AZD1208 has been shown to enhance T cell memory and improve adoptive cell therapy outcomes [42]. Among the three PIM kinases, PIM1 exhibited a positive regulation on the memory fitness of T cells [56], while PIM2 negatively modulated their anti-tumor responses [57]. The function of PIM3 in immune modulation, however, remains largely unexplored. Here, we demonstrate that both pharmacologic inhibition and genetic knockout of PIM3 enhanced memory phenotypes and cytotoxicity in CAR-T cells exposed to hypoxia. Notably, PIM3 was the only isoform significantly induced under hypoxia. Knocking out PIM3 conferred resistance to hypoxia-induced dysfunction and significantly improved CAR-T cell-mediated tumor clearance. Our findings for the first time reveal a critical role of PIM3 in modulating CAR-T metabolism and function in hypoxic conditions, providing a novel strategy for enhancing CAR-T efficacy in solid tumors via metabolic reprogramming.

While hexokinase 2 (HK2) is a hypoxia-inducible key glycolytic enzyme and inhibiting it with 2-DG has been reported to alleviate CAR-T exhaustion [58], we found that 2-DG failed to rescue the cytotoxic function of CAR-T cells under hypoxia. This aligns with findings by Hatae et al., who also reported no beneficial effects of 2-DG on hypoxic CAR-T function in vitro [47]. One possible explanation is that PIM3 inhibition downregulates multiple glycolytic enzymes, including MCT4, HK2, ENO2, PKM, TPI1, GPI, and ALDOA [42], suggesting that targeting a single enzyme like HK2 may be insufficient to reverse the metabolic dysfunction caused by hypoxia. Thus, PIM3 inhibition may be more effective due to its broader suppression of glycolysis-related pathways.

Previous approaches to counteract hypoxia in CAR-T cells have largely focused on modifying CAR constructs to become hypoxia-responsive, such as hypoxia-responsive element (HRE)-driven CAR expression or oxygen-dependent degradation domain (ODD) fusions [30, 32]. While these designs modulate CAR activity in low-oxygen conditions, our approach takes a fundamentally different route by targeting an intracellular kinase, PIM3, which is selectively upregulated under hypoxia and drives metabolic reprogramming. Inhibiting PIM3 attenuated glycolytic activity and enhanced CAR-T cell memory and cytotoxicity in hypoxic environments without altering CAR surface expression. Compared with other metabolic modulators including metformin and rapamycin [25, 47], which broadly alter cellular metabolism and may raise safety concerns due to unintended effects on non-target tissues, our strategy provides a more precise means of reinforcing CAR-T cell function under hypoxic stress, offering a novel avenue to enhance their efficacy against solid tumors.

Despite these promising results, our study has some limitations. The in vitro hypoxic model used here does not fully recapitulate the complexity of tumor hypoxia, where tumor cells, stromal components, and immunosuppressive factors interact to modulate immune responses. More advanced models, such as patient-derived organoids (PDOs), may offer a more physiologically relevant system for evaluating CAR-T performance under hypoxia. Nonetheless, our results were validated in in vivo models and were consistent with prior studies on hypoxic T cells, supporting the relevance of our approach. In addition, targeting genes upregulated under hypoxia in our study, such as prolyl 4-hydroxylase subunit alpha 1 (P4HA1), promoted T cell memory and enhanced their systemic anti-cancer immunity [59], further highlighting the potential of our model for future therapeutic discovery. Another limitation lies in the relatively short period of our in vivo experiment, which does not fully capture the long-term persistence and durability of CAR-T cells that are critical for clinical translation. Nevertheless, our data show that PIM3 KO CAR-T cells exhibit increased CD62L expression compared with WT CAR-T cells, indicating a more profound memory phenotype, which is generally associated with improved long-term functionality. Future studies incorporating longer follow-up times will be essential to confirm the durability of PIM3 KO CAR-T cells and further elucidate their translational potential.

For the broader application of our findings, several key considerations remain. First, it is essential to establish whether PIM3 inhibition can be effectively incorporated into large-scale CAR-T cell manufacturing. Given the widespread use of CRISPR/Cas9-mediated gene editing in clinical studies [60, 61], ex vivo PIM3 knockout during CAR-T cell production appears technically feasible and compatible with current Good Manufacturing Practice (GMP) workflows. Nevertheless, the potential safety concerns associated with gene editing cannot be overlooked. Off-target genetic changes introduced during editing could inadvertently alter T-cell functionality [62, 63]. To mitigate these risks, careful sgRNA design, thorough off-target screening, and rigorous functional validation of the modified CAR-T cells are required.

## Conclusions

In summary, our study demonstrates that hypoxia impairs CAR-T cell proliferation, survival, memory formation, and cytotoxicity while driving a shift toward glycolysis. We identified PIM3 as a key driver of these effects and showed that its inhibition restores CAR-T functionality by reversing hypoxia-induced metabolic dysfunction. These findings support the use of metabolic reprogramming strategies, particularly PIM3 targeting, to overcome hypoxia-related barriers and enhance CAR-T efficacy in solid tumors.

## Supplementary Information

Below is the link to the electronic supplementary material.

Supplementary Material 1

## Data Availability

All data generated or analyzed during the study are included in the paper or its online supplemental information, or available upon request.

## Abbreviations

CAR: Chimeric antigen receptor
FDA: Food and Drug Administration
TME: Tumor Microenvironment
HIFs: Hypoxia-Inducible Factors
PIM3: PIM Family Kinase 3
TNF: Tumor Necrosis Factor
IFN: Interferon
PD-1: Programmed Death-1
MSLN: Mesothelin
CD70: Tumor necrosis factor ligand superfamily member 7
PBMC: Peripheral Blood Mononuclear Cel
OCR: Oxygen Consumption Rate
LGMN: Legumain
HK2: Hexokinase 2
GBM: Glioblastoma Multiforme
PER: Proton Extrusion Rate
ECAR: Extracellular Acidification Rate
siRNA: Small Interfering RNA
