# Supplementary material for: Metabolic reprogramming through PIM3 inhibition reverses hypoxia-induced CAR-T cell dysfunction in solid tumors
Source: J Transl Med. 2025 Nov 6;23:1230. doi: 10.1186/s12967-025-07278-5 (PMC12590754; doi:10.1186/s12967-025-07278-5)
Supplement: Supplementary file 1 [file 12967_2025_7278_MOESM1_ESM.odt]

Supplementary materials

Table S1. Sequences of qPCR primers.

| Gene name | Primer sequence (5' to3' ) |
| --- | --- |
| PIM1 | F: GGCTCGGTCTACTCAGGCA |
| R: GGAAATCCGGTCCTTCTCCAC |
| PIM2 | F: TCCCGTGGAGTTGTCCATC |
| R: GGCACCAGAACCAAAATCAATG |
| PIM3 | F: CTCCAAGTTCGGCTCCCT |
| R: CGCCCAACAGTCATCAGC |
| MCT4 | F: CGGCTTTGTGCTTTACGCC |
| R: GCTGAAGAGGTAGACGGAGTA |
| HK2 | F: AGGTGGACTAAGCAATGAAGAG |
| R: CAGGAACAATAACGCATACAAG |
| LGMN | F: CGACGGGATGATTCACAGT |
| R: TTTCAAAGCAGCGACCAG |
| ENO2 | F: AGCCTCTACGGGCATCTATGA |
| R: TTCTCAGTCCCATCCAACTCC |
| PKM | F: ATGTCGAAGCCCCATAGTGAA |
| R: TGGGTGGTGAATCAATGTCCA |
| TPI1 | F: CTCATCGGCACTCTGAACG |
| R: GCGAAGTCGATATAGGCAGTAGG |
| GPI | F: CAAGGACCGCTTCAACCACTT |
| R: CCAGGATGGGTGTGTTTGACC |
| ALDOA | F: CAGGGACAAATGGCGAGACTA |
| R: GGGGTGTGTTCCCCAATCTT |

Table S2. Sequences of PIM3-targeting sgRNAs.

| sgRNA | Sequence (5' to3' ) |
| --- | --- |
| sgRNA #1 | CGGGTAGCCGCATCGCCGAC |
| sgRNA #2 | GCGGGTAGCCGCATCGCCGA |

缺氧Figures-update_08

Figure S1. Hypoxia induced metabolic reprogramming in CAR-T cells. A-D, Expression of metabolic genes involved in glycolysis (A), oxidative phosphorylation (B), fatty acid oxidation (C) and mitochondrial carrier family (D) of normoxic and hypoxic CAR-T cells. The color bar indicates normalized z-scores for each DEG. 

缺氧Figures-update_09

Figure S2. Upregulation of LGMN, PIM3, and HK2 in hypoxic CAR-T cells. A-E, Relative mRNA fold changes of indicate genes in hypoxic CAR-T cells compared with normoxic controls after 2, 4 and 6 days of treatment. All data are from at least three donors and are presented as mean ± SD. *p<0.05; **p<0.01, determined by two-way ANOVA.

缺氧Figures-update_10

Figure S3. In vitro functional validation of anti-CD70 CAR-T Cells. A, Schematic representation of the CD70 CAR construct. B, CD70 CAR expression rate in T cells on day 5 after lentiviral transduction. C, Relative mRNA fold change of PIM3 in hypoxic anti-CD70 CAR-T cells compared to normoxic controls on day 6 of treatment. D-E, Memory phenotypes (D) and apoptotic rates (E) of anti-CD70 CAR-T cells in each group following 6 days of hypoxic culture. F, Cytotoxicity assay of anti-CD70 CAR-T cells in each group after 6 days of hypoxic culture. Anti-CD70 CAR-T cells were co-cultured with U87-luc and 786-0-luc cells at an E:T ratio of 1:3 for 24 hours, and cytotoxic activity was assessed using a luciferase reporter assay. G-H, Multi-round killing assays of anti-CD70 CAR-T cells in each group targeting U87-luc and 786-0-luc cells after 6 days of hypoxic culture (E:T ratio = 1:3). All data are from at least three donors and are presented as mean ± SD. *p<0.05; **p<0.01; ***p<0.001, determined by two-tailed unpaired t-test (C), one-way ANOVA (D-E), and two-way ANOVA (F-H).

缺氧Figures-revise(1)_11

Figure S4. Knockdown efficiency and phenotypes of PIM3 KO CAR-T cells. A, Relative mRNA levels of PIM1 and PIM2 in normoxic and hypoxic anti-MSLN CAR-T cells. B, Relative mRNA fold changes of PIM3 to evaluate knockout efficiency. PIM3 KO#1, PIM3 KO using sgRNA#1; PIM3 KO#2, PIM3 KO using sgRNA#2; PIM3 KO#1+2, PIM3 KO using both sgRNA#1 and #2. C-E, Memory phenotypes of wild-type (WT) and PIM3-knockout (KO) CAR-T cells after being cultured under hypoxic conditions for 6 days were detected. The proportion CD62L-positive (C) and TTE (D) cells were calculated and representative flow cytometry plots were shown (E). F, Multi-round killing assays of hypoxia-conditioned WT and three PIM3 KO CAR-T cell lines against 1806-luc cells conducted under normoxia. All data are from at least three donors and are presented as mean ± SD. ns, not significant; *p<0.05; **p<0.01; ***p<0.001, determined by two-way ANOVA (A, F) and one-way ANOVA (B-D).

缺氧Figures-update_12

Figure S5. Safety profile of in vivo anti-tumor experiments with PIM3 KO CAR-T cells. A, HE staining results of mouse tissues and organs. B, Body weight records of mice. The data are presented as mean ± SD.
